# Prevalence and Genetic Diversity of Hepatitis B and C Viruses in People Living with HIV in the Capital District of Venezuela

**DOI:** 10.3390/v18101103

**Published:** 2026-10-06

**Authors:** Julie Andreina Beltrán, Yoneira Fabiola Sulbarán, Nahir Martínez-Urbina, Mario Comegna, María Graciela López, Lily Soto, Carlos Pérez, Moraima Hernández, Flor Helene Pujol, Rossana Celeste Jaspe

**Affiliations:** 1 Laboratorio de Virología Molecular, Centro de Microbiología y Biología Celular (CMBC), Instituto Venezolano de Investigaciones Científicas (IVIC), Caracas 1020, Venezuela; beltranjulie@gmail.com (J.A.B.); yfsulbara@gmail.com (Y.F.S.); 2 Unidad de Microscopía Electrónica, Centro de Química, Instituto Venezolano de Investigaciones Científicas (IVIC), Caracas 1020, Venezuela; 3 Sección de Virología, Instituto de Medicina Tropical, Universidad Central de Venezuela (UCV), Caracas 1050, Venezuela; nahir.martinez@ucv.ve; 4 Fundación Proyecto Once Trece, Caracas 1060, Venezuela; mcomegna20076@gmail.com; 5 Unidad de VIH, Servicio de Enfermedades Infecciosas, Hospital de Niños J. M. de los Ríos, Caracas 1011, Venezuela; magrelopez@gmail.com; 6 Sección de Inmunología, Instituto de Medicina Tropical, Universidad Central de Venezuela (UCV), Caracas 1050, Venezuela; dralilysoto@gmail.com; 7 Unidad de Infectología, Hospital Dr. Jesús Yerena, Caracas 1010, Venezuela; cpperz2000@yahoo.com; 8 Unidad de Infectología, Maternidad Concepción Palacios, Caracas 1010, Venezuela; moraima_h@yahoo.es

**Keywords:** HBV, HCV, HIV, PLWH, OBI, genotype, prevalence, IDU, PWID

## Abstract

Coinfection between hepatitis viruses and human immunodeficiency virus (HIV) significantly increases liver disease and AIDS progression. This retrospective cross-sectional study evaluated the prevalence and genetic diversity of hepatitis B (HBV) and C viruses (HCV) among people living with HIV (PLWH) in Venezuela’s Capital District. A total of 420 serum samples collected between 2022 and 2025 were tested for serological and molecular markers, including whole-genome HBV sequencing via next-generation sequencing (NGS) to characterize viral genotype distribution and mutations. Seroprevalences for HBsAg, anti-HBc, and anti-HCV were 2.9%, 20%, and 4.0%, respectively. HBV exposure was significantly higher in older men, with no differences between treatment-naïve and treated patients. Occult HBV infection (OBI) prevalence decreased compared to 2014 data (seropositive: 7% vs. 16%, seronegative: 0.9% vs. 20%). This decline, along with the higher HBV exposure among older adults, may be partially attributed to the universal HBV vaccination introduced in Venezuela in 1998, as well as modifications in antiretroviral therapy affecting HBV replication. Notably, key vaccine- and diagnostic-escape mutations were detected in OBI cases, highlighting clinical and public health concerns. Increased HCV prevalence appears to be linked to rising intravenous drug use in the country. HBV phylogenetic analysis revealed genotype F3 as predominant, followed by F1b (Cosmopolitan clade), while HCV showed genotype 1a to be the most frequent, followed by 1b and 3a. These findings underscore the success of immunization efforts but highlight the urgent need for continuous molecular surveillance and updated public health policies, including targeted harm-reduction programs, to control viral hepatitis in vulnerable populations.

## 1. Introduction

Human immunodeficiency virus (HIV), one of the most common blood-borne viruses, shares overlapping transmission routes with hepatotropic viruses, hepatitis B virus (HBV), and hepatitis C virus (HCV) [1]. The coinfection of HIV with HBV or HCV constitutes a major public health concern worldwide [2]. According to the latest World Health Organization (WHO) Global Hepatitis Report 2026, viral hepatitis caused approximately 1.3 million deaths in 2024, remaining the second leading infectious cause of death globally. In addition, an estimated 287 million people have been living with chronic hepatitis B or C infection this year, with the majority of deaths attributed to HBV [2]. The burden of these infections is compounded by the fact that more than 39 million people live with HIV (PLWH) [2].

With the advent of effective antiretroviral therapy (ART), the life expectancy of PLWH now approaches that of the general population [2]. Consequently, the relative importance of non-AIDS-related morbidity has increased in recent years. After AIDS-related deaths and non-AIDS-defining cancers, liver disease is the third leading underlying cause of death among PLWH [3]. Chronic viral hepatitis has emerged as a significant cause of morbidity and mortality among PLWH [4].

HIV–hepatitis virus coinfection accelerates liver disease progression and is associated with an elevated risk of death and increased occurrence of AIDS-associated complications. In patients with HIV, HBV and HCV infections carry an increased risk of chronicity, resulting in higher viral loads and a reduced chance of spontaneous viral clearance. Epidemiological evidence suggests that HIV coinfection may increase the risk of cirrhosis and hepatocellular carcinoma (HCC) in patients with HBV or HCV [5,6]. Furthermore, HIV-HCV coinfection has been associated with higher rates of HCV persistence, higher viral loads, accelerated progression of hepatic fibrosis, and development of end-stage liver disease than HCV mono-infection [6,7]. A global systematic review estimated that 7.4% of PLWH are co-infected with HBV and 6.2% are co-infected with HCV [7]. HBV-HIV coinfection rates are geographically heterogeneous, ranging from 5% to 20% worldwide, depending on factors such as the distribution of risk groups, implementation of HBV vaccination programs, and level of endemicity in the general population [8]. Meanwhile, the highest HCV-HIV coinfection prevalence has been observed among people who inject drugs (PWIDs) (82.4%) [9]. In resource-limited settings, such as Latin America, regional disparities in access to ART and routine molecular diagnostics severely constrain surveillance efforts. This creates a critical diagnostic gap, as standard screening relies predominantly on surface antigen detection, overlooking the underdiagnosed entities.

Among these, HBV can manifest as either overt or occult infection. Occult HBV infection (OBI), defined as the presence of HBV DNA in the liver (with detectable or undetectable serum HBV DNA) in the absence of HBsAg, represents a diagnostically challenging form of HBV infection that may also lead to chronicity and liver complications [10]. From a serological standpoint, OBI is subclassified into seropositive OBI, when individuals are HBsAg-negative but anti-HBc and/or hepatitis B surface antibody (anti-HBs)-positive, and seronegative OBI, in individuals who test negative for all three markers, with the latter being particularly elusive to conventional screening and thus requiring molecular methods for reliable diagnosis [10]. The presence of vaccine-escape mutations can further complicate diagnosis and prevention efforts [11]. In PLWH, the development of these mutations is often favored by altered host immune pressure and selective pressure exerted by low adherence to antiretroviral therapy with dual activity against HIV and HBV, such as lamivudine (3TC) and tenofovir, included in fixed-dose combinations such as TLD (TDF; tenofovir disoproxil fumarate, 3TC, and dolutegravir). Overlapping polymerase and surface gene reading frames mean that drug-resistance mutations can simultaneously alter HBsAg antigenicity, potentially yielding vaccine-escape or diagnostic-escape variants [12].

The distribution of HBV and HCV genotypes varies geographically and has important implications for disease progression, treatment response, and vaccine efficacy [4,13,14]. In Latin America, a region characterized by diverse ethnic admixture and migratory patterns, HBV genotypes F and H are considered autochthonous, with genotype F being the most prevalent and divergent in South America [15]. Genotype F is subdivided into six subgenotypes (F1–F6), reflecting distinct geographic clustering and evolutionary trajectories [15,16]. In Venezuela, subgenotype F3 is considered the predominant variant in the general population, followed by F2 [17]. Eight genotypes and over 100 HCV subtypes have been described. Globally, genotype 1 (mainly subtypes 1a and 1b) is the most common genotype, including in South America [14]. In Venezuela, G1 is the most common genotype, followed by G2 and G3 [18].

Although HBV and HCV coinfections among PLWH in Venezuela have been previously documented [19], updated molecular surveillance remains scarce. The prolonged socioeconomic and healthcare crises in the country have compromised routine screening and vaccination coverage, potentially exacerbating high-risk behaviors such as intravenous drug use (IDU) [20]. Furthermore, nearly three decades after Venezuela established universal HBV vaccination in 1998, evaluating its long-term impact on viral prevalence among young adults is imperative. Therefore, this study aimed to evaluate the prevalence and circulating genotypes of HBV and HCV in PLWH in the Capital District of Venezuela, providing current epidemiological evidence to guide prevention, diagnosis, and treatment strategies for this vulnerable population.

## 2. Materials and Methods

### 2.1. Blood Samples

A total of 420 blood samples were collected from PLWH in Venezuela between 2022 and 2025 after obtaining written informed consent. Serum samples were stored at −80 °C until use. The cohort comprised 293 males (70%) and 127 females (30%), with a mean age of 36.5 years (range: 10 months to 74 years; SD ± 13.8). Participants were classified as treatment-naïve (*n* = 246) or on/with a history of antiretroviral therapy (*n* = 174). Most treated patients (83.8%, *n* = 134/160 for whom ART information could be confirmed) were receiving a fixed-dose combination of TLD as ART. Among participants with available information regarding IDU, 3.7% (14/378) were PWIDs (12 males and 2 females) with a mean age of 35.9 years (range: 18 to 58 years; SD ± 11.4). The study population included 33 children and adolescents (aged 10 months to 17 years) under the care of the Infectious Diseases Unit at J. M. de los Ríos Children’s Hospital. Most participants resided in the Capital District. The Bioethics Committee of the Instituto Venezolano de Investigaciones Científicas (IVIC) approved this study (ethical approval code: CD-2024/0874).

### 2.2. HBV and HCV Serologic Markers

All serum samples were tested for Hepatitis B surface antigen (HBsAg) using a Monocent Immunoenzymatic assay (ELISA) kit (Monocent Inc., Los Angeles, CA, USA), total antibodies against the hepatitis B core antigen (anti-HBc) using a Dia.Pro kit (Dia.Pro Diagnostic Bioprobes S.r.l., Sesto San Giovanni, Italy), and antibodies against HCV (anti-HCV) using a Monocent ELISA kit (Monocent Inc., Los Angeles, CA, USA), according to the manufacturer’s instructions. Samples that tested positive for HBsAg and anti-HBc were further evaluated for the presence of hepatitis B e antigen (HBeAg) and antibodies against it (anti-HBe) using a DIASource kit (DIAsource ImmunoAssays/Dia. Pro Diagnostic Bioprobes S.r.l., Sesto San Giovanni, Milan, Italy). Absorbance was determined using a SpectraMax250 ELISA reader (Molecular Devices, San Jose, CA, USA) at 450 nm with a secondary filter of 620–630 nm.

Samples showing absorbance values above or near the cut-off value were re-tested using a second third-generation ELISA, according to the manufacturer’s instructions (RecombiLISA, TK Biotech, Inc., Poway, CA, USA).

### 2.3. HBV DNA Detection and Sequencing

Total DNA was extracted from 194 serum samples, including 12 HBsAg-positive samples, 72 HBsAg-negative/antiHBc-positive samples, and 110 randomly selected HBsAg-/anti-HBc-negative samples. Seronegative samples were selected via simple random sampling from all available seronegative individuals in the cohort who met the minimum volume requirement for extraction (≥500 µL), ensuring an unbiased representative baseline group. These randomly selected samples comprised 73 males (66.4%) and 37 females (33.6%), with a mean age of 36.2 years (SD ± 12.3), thereby preserving the age distribution and sex proportions of the overall study population. DNA extraction was performed using the QIAamp DNA Mini Kit (QIAGEN GmbH, Hilden, Germany), according to the manufacturer’s instructions. HBV DNA was identified by nested PCR amplification of the S gene, as previously described [19]. The first round employed primers 58p (sense: 5-CCTGCTGGTGGCTCCAGTTC-3) and 1101n (antisense: 5-GAAAGGCCTTGTAAGTTGGCGAG-3), generating a 1043 bp product (nt 58–1101). Two independent second-round amplifications (S6 and S3) were performed using the primers S6 (sense: 5-GCACACCGGGATCCGAGGACTGGGGACCCT-3) or S3 (sense: 5-TGCCTCATCTTCTTRTTGGTTCT-3) together with S3as (antisense: 5-CCCCAAWACCAVATCATCCATATA-3), yielding products of 628 bp and 338 bp, respectively. Amplification rounds were performed using Platinum™ Taq DNA Polymerase (Thermo Fisher Scientific, Waltham, MA, USA) under the following conditions: 94 °C for 3 min; 39 cycles of 94 °C for 45 s, 45 °C for 45 s, and 72 °C for 60 s; and a final extension at 72 °C for 7 min. The second round of PCR was performed under the following conditions: 95 °C for 10 min; 35 cycles of 95 °C for 20 s, 55 °C for 20 s, and 72 °C for 45 s; and a final extension at 72 °C for 5 min.

All amplicons of the expected size were purified and bidirectionally sequenced using 10 pmol of sense and antisense inner primers through the sequencing service provided by Macrogen, Inc. (Seoul, Korea). The partial S gene sequences were deposited in GenBank under accession numbers PZ863007–PZ863009.

### 2.4. HBV Viral Load Quantification

HBV viral load was determined in all samples that tested positive for anti-HBc, as well as in serologically negative samples that tested positive by PCR for the S6 and/or S3 regions, using a commercial HBV Real-TM Quant Dx kit (Sacace Biotechnologies S.r.l., Como, Italy) according to the manufacturer’s instructions. The limit of detection (LOD) and lower limit of quantification (LOQ) for this assay were 7 IU/mL (using the 1.0 mL sample preparation procedure), with a dynamic range up to 10^8^ IU/mL.

### 2.5. Full-Length HBV Genome Sequencing by Next Generation Sequencing (NGS)

The full-length 3.2 kb HBV genome was amplified using the primers P1 (sense: 5′-CCGGAAAGCTTGAGCTCTTCTTTTTCACCTCTGCCTAATCA-3′) and P2 (antisense: 5′-GGTCGTGGTACGTTGAAAAACTTCTCGAGTTCGAAAGGCC-3′), as described by Günther et al. [21], and using F1813 (sense: 5′-CCAKCACCATGCAACTTTTTCACCTC-3′) and R1780 (antisense: 5′-GMACAGACCMATTTATGCCTACAGC-3′) primers, as described by Cremer et al. [22]. In cases where full-genome amplification was not achieved, overlapping fragments of 700–1400 bp were generated using highly conserved primers in a second round starting from the first round P1–P2 [21], with the following primer combinations: 58p-1450n (1392 bp) (sense: 5′-CTGCTGGTGGCTCCAGTTC-3′) (antisense: 5′-GATTCAGCGCCGACGGGACGTA-3′), 1101p-P2 (1392 bp) (sense: 5′-CTCGCCAACTTACAAGGCCTTTC-3′), P1-2853n (1032 bp) (antisense: 5′-TGTTCCCAAGAATATGGTGA-3′), and 2440p-409n (1180 bp) (sense: 5′-GCCGCGTCGCAGAAGATCTCAA-3′) (antisense: 5′-AGATGAGGCATAGCAGCAGGATG -3′). The first-round conditions were as follows: 94 °C for 30 s; 36 cycles of 94 °C for 16 s, 60 °C for 30 s, and 68 °C for 3,15 min; and final extension at 68 °C for 5 min. The second-round conditions were as follows: 94 °C for 1 min; 43 cycles of 94 °C for 45 s, 45 °C for 60 s, and 72 °C for 60 s; and a final extension at 72 °C for 7 min. Amplicons were visualized by electrophoresis on a 2% (*w*/*v*) agarose gel.

Approximately equimolar amounts of each amplicon were pooled to generate sequencing libraries. Library preparation was performed using the Microbial Amplicon Prep Kit (iMAP) with IDT for Illumina-PCR Indexes set 1–3 (Illumina, San Diego, CA, USA), following the manufacturer’s instructions. Libraries were pooled, quantified using the Qubit DNA HS assay (Thermo Scientific, Waltham, MA, USA), and sequenced on an iSeq 100 platform using a 300-cycle V2 kit (Illumina, San Diego, CA, USA) with paired-end sequencing, 10% PhiX control v3, and a loading concentration of 50 pM. Viral genome assembly was performed using the Genome Detective Virus Tool Version 2.22.2 (https://www.genomedetective.com/) [23]. The complete genome nucleotide sequences were deposited in GenBank under the accession numbers QB060664–QB060672.

### 2.6. Analysis of Mutations in the HBV Genome

Mutation analysis of the S gene, which encodes the surface protein and may lead to vaccine-escape mutants or immunoassay detection failure, was performed using the Geno2pheno platform developed by the Max Planck Institute for Informatics (https://hbv.geno2pheno.org/index.php, accessed on 20 July 2026) [24]. Mutations within the “a” determinant and major hydrophilic region (MHR) (amino acid positions 100–147) of HBsAg were analyzed. Owing to the overlap between the S gene and the polymerase (P) gene open reading frames, mutations conferring resistance to antiviral drugs (Adefovir, Entecavir, Lamivudine, Telbivudine, and Tenofovir) were examined in the P gene using both the Geno2pheno platform [24] and the Hepatitis B Virus Database of Lyon (https://hbvdb.lyon.inserm.fr/HBVdb/, accessed on 24 November 2025) [25].

In cases where the complete HBV genome sequence was obtained, the Geno2pheno[hbv] platform [24] was additionally employed to evaluate clinically relevant amino acid substitutions across the pre-core and core proteins.

### 2.7. HCV RNA Detection and Genotyping

HCV RNA was detected by amplifying the 5′ non-coding region (5′NC) using reverse transcription and nested polymerase chain reaction (RT-PCR), as previously described [19]. Genotyping was performed by amplifying the NS5B region [19]. In both cases, the first round of amplification was performed using the SuperScript III One-Step RT-PCR System with Platinum Taq High-Fidelity DNA Polymerase, and the second round of PCR was performed using Platinum™ Taq DNA Polymerase (Thermo Fisher Scientific, Waltham, MA, USA).

The first round of 5′NC amplification employed primers 939P (sense: 5-CTGTGAGGAACTACTGTCTT-3) and 209N (antisense: 5′-CTGTGAGGAACTACTGTCTT-3′), generating a 300 bp product. The second round of amplification was performed using primers 940P (sense: 5′-TTCACGCAGAAAGCGTCTAG-3′) and 211N (antisense: 5′-TGACGGACTATCCCACGAGCTCTCAC-3′), yielding a 258 bp product. The first-round RT-PCR conditions were as follows: 50 °C for 20 min, 94 °C for 5 min, 24 cycles of 94 °C for 20 s, 50 °C for 20 s, and 68 °C for 30 s, and a final extension at 68 °C for 5 min. The second-round conditions were as follows: 94 °C for 5 min; 24 cycles of 94 °C for 20 s, 50 °C for 20 s, and 72 °C for 30 s; and a final extension at 72 °C for 5 min.

The first round of NS5B region amplification employed primers 8245P (sense: 5′-TGGGGATCCCGTATGATACCCGCTGYTTYGA-3′) and 8645N (antisense: 5′-AAGTGCCTCCGATACTGGTCCTTAAGGCGG-3′), which generated a 400 bp product. The second round of amplification was performed using primers 8276P (sense: 5-CTCCACAGTCACTGAGAGCGACAT-3) and 8645N (antisense: 5-AAGTGCCTCCGATACTGGTCCTTAAGGCGG-3), yielding a 346 bp product. The first-round conditions were as follows: 50 °C for 30 min; 94 °C for 3 min; 35 cycles of 95 °C for 30 s, 52 °C for 30 s, and 72 °C for 45 s; and a final extension at 75 °C for 5 min. The second-round conditions were as follows: 94 °C for 4 min; 35 cycles of 94 °C for 30 s, 50 °C for 30 s, and 72 °C for 45 s; and a final extension at 72 °C for 7 min.

All amplicons of the expected size were purified and bidirectionally sequenced using 10 pmol of sense and antisense inner primers through the sequencing service provided by Macrogen, Inc. (Seoul, Korea). The resulting sequences were deposited in GenBank under accession numbers PZ862998–PZ863006 and PZ862989–PZ862997.

### 2.8. HCV Viral Load Quantification

HCV viral load was determined in all samples with positive anti-HCV serology using a commercial HCV Quantification kit (Bosphore, Anatolia GeneWorks, Istanbul, Turkey) according to the manufacturer’s instructions. The assay features a lower limit of detection (LOD) of 13.5 IU/mL and a lower limit of quantification (LOQ) of 25 IU/mL (2.5 × 10^1^ IU/mL), with a dynamic range extending up to 10^8^ IU/mL.

### 2.9. Phylogenetic and Sequence Analysis

Sequence alignments were performed using MAFFT version 7 [26]. Phylogenetic analyses were performed using the Maximum Likelihood method with IQ-TREE multicore version 2.3.4 (https://iqtree.github.io/, accessed on 18 August 2026) [27], and with 1000 or 5000 bootstrap replicates. The best-fit substitution model was selected using ModelFinder as implemented in IQ-TREE version 2.3.4. Reference sequences from different genotypes and subgenotypes were included in the phylogenetic analysis, along with closely related sequences obtained via BLAST analysis version 2.17.0 (https://blast.ncbi.nlm.nih.gov/, accessed on 18 August 2026) [28]. Phylogenetic trees were visualized and annotated using iTOL version 7.4.2 (https://itol.embl.de/, accessed on 18 August 2026) [29].

### 2.10. Statistical Analysis

Bivariate comparisons were evaluated using the chi-square test with Yates’ correction or Fisher’s exact test when any expected value was less than 5. Independent predictors (sex, age, treatment status, and IDU condition) were assessed using multivariate logistic regression analysis in a subgroup of 378 PLWH. Adjusted odds ratios (aORs) with 95% confidence intervals (95% CIs) were reported, and *p*-values < 0.05 were considered statistically significant. All analyses, including multivariable logistic modeling, were performed using Epi Info software version 7.2.7.0 (Centers for Disease Control and Prevention, Atlanta, GA, USA) [30].

## 3. Results

### 3.1. Prevalence of HBV and HCV in PLWH in Venezuela

Serological markers for HBV and HCV were determined in samples from 420 PLWH in Venezuela, collected between 2022 and 2025 (Table 1). Participants were stratified based on their ART status as either treatment-naïve (*n* = 246) or on treatment, with the latter representing individuals currently receiving or having a history of ART (*n* = 174) (Figure 1). The overall prevalence rates of HBsAg, anti-HBc, and anti-HCV were 2.9%, 20%, and 4.0%, respectively (Figure 1). No statistically significant association was observed between ART status and seropositivity for HBsAg (3% vs. 3%, *p* = 0.986, chi-square test; aOR = 0.73, 95% CI: 0.20–2.67), anti-HBc (18% vs. 21%, *p* = 0.449, chi-square test; aOR = 0.76, 95% CI: 0.44–1.32), or anti-HCV (4% vs. 4%, *p* = 0.983, chi-square test; aOR = 1.10, 95% CI: 0.38–3.22) (Figure 1).

In the total cohort of 420 PLWH, the mean age was 36.5 years (range: 10 months to 74 years; SD ± 13.8 years). Among the patients, 127 (30%) were female (mean age: 35.5 years), and 293 (70%) were male (mean age: 37.0 years). This group also included 33 children and adolescents (aged 10 months to 17 years old). In the multivariable logistic regression model, age was identified as the sole independent predictor of HBsAg reactivity, with the odds of positivity increasing by 7.0% for each additional year of age (aOR = 1.07, 95% CI: 1.02–1.13, *p* = 0.0116). Regarding sex, the prevalence of HBsAg was higher in males between 51 and 70 years of age, without any significant differences between sexes (1% in females vs. 4% in males, *p* = 0.117, Fisher’s exact test, aOR = 3.98, 95% CI = 0.49 − 32.50). The prevalence of total anti-HBc was significantly higher in males (24%, 69/293) than in females (10%, 13/127) (*p* = 0.002, chi-square test), with a higher prevalence in individuals aged 61–71 years (Table 1). In the multivariable logistic regression analysis, both age and male sex remained independent predictors of anti-HBc seropositivity. Specifically, each one-year increase in age was associated with a 6.2% increase in the odds of anti-HBc reactivity (aOR = 1.06, 95% CI: 1.04–1.09), while male participants had 2.5-fold higher odds of seropositivity compared to females (aOR = 2.52, 95% CI: 1.28–4.97). Anti-HCV was positive in 2% (3/127) of females and 5% (14/293) of males, without any significant sex differences (*p* = 0.295, Fisher’s exact test; aOR = 1.62, 95% CI: 0.44–5.99). In both groups, the highest prevalence was observed in individuals aged 51–60 years, although age was not significantly associated with positivity in the multivariable model (aOR = 1.01 per additional year, 95% CI: 0.97–1.06, *p* = 0.492). In a subgroup of patients with known status regarding intravenous drug use (IDU; *n* = 378/420), 3.7% (14/378) were identified as PWIDs. Among these 14 individuals, four had HCV and one had overt HBV infection. Notably, the prevalence of anti-HCV was significantly higher among PWID (4/14, 28.6%) compared to non-PWIDs (13/364, 3.6%), with IDU remaining the sole independent risk factor strongly associated with anti-HCV seropositivity in the multivariable logistic regression analysis (aOR = 11.06, 95% CI: 2.99–40.98, *p* = 0.0003). In contrast, IDU was not significantly associated with HBV serological markers in the multivariable models, showing no independent relationship with either overt infection (HBsAg: aOR = 2.89, 95% CI: 0.31–26.57, *p* = 0.349) or past exposure (anti-HBc: aOR = 2.39, 95% CI: 0.72–7.97, *p* = 0.156). No serological evidence of HBV or HCV infection was observed in the pediatric and adolescent populations (0–17 years). The prevalence of HBV exposure (anti-HBc) was higher than that of HCV, whereas the prevalence of active HBV infection (HBsAg) was lower (Figure 1).

HBV DNA was detected by PCR amplification of the surface gene region in nine of the 12 HBsAg-positive samples; notably, two of these nine were positive only by qPCR (B1322 and B5580), exhibiting low viral load. One (B5580) received additional entecavir therapy (Table 2). Among these nine samples, eight were anti-HBc-positive, and six were HBeAg-positive. The remaining patient was anti-HBc-negative but HBeAg-positive and anti-HBeAg-negative (BLC97), a pattern suggestive of acute infection. All three HBsAg-positive individuals with undetectable HBV DNA were older than 49 years and were receiving a TLD-based treatment regimen, which exerts dual activity against HIV and HBV. This dual activity might explain the lack of amplification and undetectable viral loads (BLC110, BLC98, and B5600 samples; Table 2). HBV viral loads in individuals with overt (HBsAg-positive) infection ranged from 3.47 × 10^1^ to 1.43 × 10^8^ IU/mL (Table 2).

HCV RNA was detected in 10 of the 17 anti-HCV-positive samples, either by RT-PCR amplification of the 5′NC and NS5B regions or by viral load determination, with no differences observed according to ART status. The HCV viral loads in these individuals ranged from 4.53 × 10^2^ to 7.07 × 10^5^ IU/mL.

### 3.2. Occult Hepatitis B Infection (OBI) Prevalence

Occult HBV infection (OBI) is characterized by HBV infection without detectable HBsAg in the serum [12]. OBI was analyzed in 182 serum samples from Venezuelan PLWH (72 HBsAg-negative/anti-HBc-positive and 110 randomly selected from the 336 HBsAg-/anti-HBc-negative). Seropositive OBI (anti-HBc-positive; *n* = 72) among PLWH was detected in 7% (5/72) of samples screened using both PCR and viral load determination (Figure 2). No significant association was found between seropositive OBI prevalence and treatment status, with rates of 5% (2/39) in treatment-naïve individuals and 9% (3/33) in those undergoing treatment (*p* = 0.655, Fisher’s exact test) (Figure 2). For seronegative OBI infection (HBsAg- and anti-HBc-negative; *n* = 110), all samples were screened by PCR, with viral load determination performed strictly as a reflex test on PCR-positive samples. Overall seronegative OBI prevalence was 0.9% (1/110) in samples, with a prevalence of 1% (1/88) in treatment-naïve patients (Figure 2).

No significant difference was observed in OBI prevalence according to sex: HBV DNA was detected in the sera of 1/49 females and 5/133 males (*p* = 1.000, Fisher’s exact test). The viral loads in most OBI cases were consistent with those reported in the literature for this type of infection (<200 IU/mL). Specifically, five of the six cases exhibited viral loads ranging from 5.38 × 10^1^ to 1.56 × 10^2^ IU/mL, while only one individual showed a viral load slightly above this threshold (3.06 × 10^2^ IU/mL) (Table 3). The majority of patients (4/6) were negative for the e antigen (HBeAg and anti-HBe) (Table 3).

### 3.3. HBV and HCV Genotype Distribution in Venezuelan PLWH

To determine the genetic diversity of HBV among Venezuelan PLWH, 12 partial sequences of the HBV S gene (from 7/9 overt infections and 5/6 occult infections) and nine complete genomes (from 7/9 overt infections and 2/6 occult infections) were analyzed in this study. Phylogenetic analysis showed that genotype F3 was the most prevalent, followed by F1b and A2 (Table 4). The low viral load in occult infections hampered the complete amplification of the HBV genome in some isolates. Overt infection was predominantly associated with genotype F3 and was found to cluster with HBV isolates from Venezuelan individuals in previous studies, including Venezuelan Indigenous individuals and individuals living with HIV in Panama. In occult infections, two samples were of genotype F1b, two were A2, and one was of genotype F3 (Table 4, Figure 3).

The genotype A2 isolates from this study were related to those from the United States and Belgium (Figure 3). Two phylogenetically distinct clusters of subgenotype F1b have been described: the Basal and Cosmopolitan clades. These clusters exhibit restricted geographical distributions, with a notable epidemiological distinction: the Basal cluster is predominantly found in regions characterized by high HCC incidence, whereas the Cosmopolitan cluster is typically associated with areas of low HCC incidence [31,32]. In this context, the F1b isolates obtained in this study clustered with those from Argentina and Chile, belonging to the Cosmopolitan clade, exhibiting a bootstrap value of 100% (Figure 3). These isolates carried the differential signature pattern of eight nucleotides distributed throughout the genome, which is characteristic of the Cosmopolitan clade (G843, T2480, A2633, and C2771 in the polymerase; C1966 in pre-core; C2125 and G2200 in core; and C1688 in the X gene) [31,32]. Furthermore, it has been well established that the Cosmopolitan and Basal clusters of subgenotype F1b exhibit significant differences in the frequency of basal core promoter (BCP) and pre-core mutations, with the Basal cluster presenting substantially higher rates of the A1762T/G1764A and G1896A mutations, both of which are associated with severe liver disease [31]. Consistently, none of the F1b isolates identified in this study harbored these mutations.

In contrast, among the remaining complete genomes analyzed, isolate B1179 (genotype A2) carried the BCP A1762T/G1764A double mutation. This isolate exhibited an OBI phenotype with low viremia (1.56 × 10^2^ IU/mL) and an HBeAg-negative profile. Notably, this isolate did not harbor the C1653T and T1753V mutations, which are also present in the BCP and, in conjunction with the absence of HBeAg, reduce the risk of HCC development [13].

An analysis of 66 critical amino acid positions within HBsAg (residues 100–147), encompassing the major hydrophilic region corresponding to the “a” determinant involved in anti-HBs binding, was performed. Mutations associated with escape from HBsAg detection by diagnostic immunoassays, as well as those potentially affecting vaccine-induced protection [29,30], were identified in two of the six OBI isolates under evaluation (144A in B1058 and 120Q in B1304) and in one of the seven isolates derived from patients with overt HBV infection (120Q in B5262) (Table 5). None of the isolates showed reactivity with either of the two HBsAg ELISA kits employed in this study. However, in the isolate corresponding to the overt infection, HBsAg was detected using the ARIA HBV-5 Rapid Test immunochromatographic assay (CTK Biotech, Inc., Poway, CA, USA). Notably, this sample exhibited a significantly higher viral load than the OBI sample sharing the same mutation (Table 5). In contrast, isolate B5450 (overt infection) was found to carry the 118K mutation, which has also been previously linked to diagnostic escape (IDE-VE); nevertheless, HBsAg was successfully detected by both ELISA formats used (Monocent Inc., Los Angeles, CA, USA and RecombiLISA, TK Biotech, Inc., Poway, CA, USA ELISA kits). This suggests that although 118K is structurally recognized as an IDE-VE mutation, its phenotypic expression on diagnostic failure might be overridden in clinical samples with high HBV viral loads and/or using modern multi-epitope commercial ELISA assays, which might minimize the impact of single amino acid substitutions on HBsAg detection.

Substitutions related to resistance to entecavir, adefovir, lamivudine, telbivudine, or tenofovir [24,25,33] were not detected in any of the HBV isolates examined in the present study. Three of the four patients reported discontinuing ART; additionally, while the youngest patient was confirmed to be vaccinated against HBV, the remaining three were presumed to be unvaccinated.

The HCV genotype distribution showed that genotype 1a (7/9, 78%) was the most prevalent, with nucleotide sequence identities among the samples of this study ranging from 91.5% to 97.0%. This was followed by genotype 1b (1/9, 11%), which showed 95.9–96.3% identity between the sample from this study and the three closest sequences available in GenBank, and genotype 3a (1/9, 11%), which showed 96.4–96.7% identity between the sample from this study and the three most closely related GenBank sequences (Figure 4). Notably, among this group, four individuals reported a history of IDU: three had genotype 1a (C1269, C1328, and C1338), while the remaining one harbored genotype 3a (C1328).

## 4. Discussion

HBV and HCV share the same transmission routes as HIV. Hepatitis virus–HIV coinfection can increase the risk of hepatic fibrosis, cirrhosis, hepatocellular carcinoma, and compromise ART efficacy [4,6,7,34]. In this study, we evaluated the prevalence and circulating genotypes of HBV and HCV in 420 individuals living with HIV in Venezuela between 2022 and 2025. The prevalence of HBsAg (active infection) was 2.9%, similar to the 3.1% reported in 2014 for the same cohort [19]. Compared to the global HIV-positive populations, this prevalence was lower than that documented in the Americas and the Caribbean (5–7%) [35,36]. The prevalence of anti-HBc (exposure) was 20%, which is significantly higher than the 14% reported in 2014 (*p* = 0.036) [19]. However, compared to other South American HIV cohorts, our exposure prevalence was lower than that in Chile (43%) [37] and Brazil (37%) [38]. Anti-HBc positivity was significantly associated with older age groups (>41 years; *p* = 0.000005), consistent with previous Venezuelan reports [19] and findings from Colombia [39] and Brazil [40]. These results indicate that while HBsAg prevalence has remained stable over the past decade, HBV exposure has significantly increased in older adults.

The overall seropositive OBI prevalence (7%) in the PLWH cohort evaluated in this study was comparable to that reported in 2009 among chronic HIV-1 patients living in the urban areas of Venezuela (7%) [41], but lower than the rates observed in Venezuelan treatment-naïve PLWH in 2014 (16%) [19], global prevalence in HIV-positive patients (16%) [42], and HIV cohorts from other Latin American countries (14–49%) [43]. No significant association with antiretroviral treatment status was found. In contrast, in this study, seronegative OBI was detected in only 0.9% of the cases, which was significantly lower than the 20% previously described in treatment-naïve Venezuelan PLWH (*p* = 0.0001) [19].

The decline in OBI prevalence alongside the higher anti-HBc seroprevalence among older adults may be partially attributed to the introduction of the universal HBV vaccination program in Venezuela in 1998 [44,45]. Although individual vaccination records were unavailable and vaccination status could not be directly confirmed, the younger group of PLWH (aged less than 30 years) was born during or after the implementation of the three-dose schedule. Global modeling supports this trend, estimating that approximately 87% of infants worldwide received the three-dose HBV vaccine by 2016, contributing to the decline in HBV prevalence among younger populations [46]. However, differences in OBI prevalence between the two studies may also be attributed to additional factors. Variations in diagnostic sensitivity or sample selection between the 2014 and 2025 cohorts might also account for these differences. Beyond vaccine-derived immunity, widespread tenofovir-based ART, such as TLD, established as the regular ART regime since 2018 [20], provides robust dual anti-HIV/HBV suppression, preventing active viral replication and occult presentation. In this context, tenofovir-sparing antiretroviral therapy (ART) and pre-exposure prophylaxis (PrEP) for HIV are increasingly used; nevertheless, tenofovir-containing regimens remain uniquely advantageous due to their dual protective efficacy against both HIV transmission and incident HBV infection or reactivation [47].

In this study, three HBsAg escape mutations, T118K, P120Q, and D144A, were identified across four isolates, two of which corresponded to OBI. It is interesting to note that three out of these four cases correspond to individuals who interrupted their treatment. These amino acid substitutions within the ‘a’ determinant loop are well known to induce conformational changes that compromise antibody recognition, leading to vaccine failure and diagnostic escape [11,48,49], potentially accounting for the lack of HBsAg detection across the three different assays, including ELISA and immunochromatography. Notably, among the two isolates carrying the 120Q mutation, the one corresponding to overt HBV was reactive in only one of the three immunodiagnostic assays employed, likely attributable to the mutation itself. In contrast, the isolate associated with occult infection remained undetectable across all three assays. A plausible explanation for this differential reactivity lies in the significantly higher viral load observed in the overt case, which could have facilitated HBsAg detection despite the presence of the escape variant. In line with this pattern, although 118K is widely recognized as an immune and diagnostic escape variant, the sample harboring this mutation was successfully detected by both ELISA assays, suggesting that elevated viral titers or multi-epitope commercial formulations can offset false-negative phenotypes. Regarding D144A, its presence in an OBI sample further reinforces the role of major ‘a’ determinant alterations in HBsAg non-detection under low viral replication. To the best of our knowledge, this is the first report describing these HBsAg escape mutations in PLWH in Venezuela. Globally, the 118K mutation has been previously described in a cohort of immunosuppressed patients with HBV reactivation in Italy [50]. Similarly, the P120Q mutation has been documented in blood donors with OBI in China [51]. Finally, the 144A mutation has been described in HIV-positive individuals in Brazil [52], blood donors with OBI in China [49], and patients with chronic HBV infection in Egypt [53]. No antiviral resistance-associated mutations were detected [24,25] against entecavir, adefovir, lamivudine, telbivudine, and tenofovir. This finding is clinically relevant given that most treated patients were receiving a TLD-based regimen (tenofovir disoproxil fumarate, lamivudine, and dolutegravir). The absence of 3TC- and TDF-resistance mutations confirms that dual-active ART remains effective and that treatment selection pressure has not led to resistant HBV variants in this cohort.

The detection of escape mutations in both overt and occult HBV infections raises important diagnostic concerns. The 120Q and 144A mutations, associated with immune and diagnostic escape, may lead to underdiagnosis of HBV in PLWH, particularly in the context of OBI, where HBsAg is undetectable. This underscores the critical importance of incorporating molecular testing (PCR) into routine diagnostic algorithms for HBV in PLWH, as serological testing alone may miss a significant proportion of individuals with the disease. Enhanced surveillance of vaccine-escape variants is warranted to monitor the effectiveness of current vaccination strategies and to inform potential vaccine updates.

In Venezuela, HBV genotype F is considered the predominant genotype, with F3 being the main subgenotype; however, the circulation of genotypes F1b, F2, A, C, D, and G has also been documented [17,19,54,55]. In the present study, phylogenetic analysis of HBV isolates from PLWH with overt (HBsAg-positive) infection confirmed that genotype F3 remains the most prevalent in this population, followed by F1b and A2 [19]. Notably, this genotype distribution differed from that observed recently in Venezuelan patients with HBV mono-infection: although F3 was the most common subgenotype in both groups, F2 was the second most prevalent in mono-infection [17,19], whereas F1b ranked second in the PLWH. This is similar to the findings reported in 2014 [19]. The F1b samples characterized in this study were phylogenetically related to Argentinian and Chilean strains within the F1b Cosmopolitan clade. Notably, this sublineage is associated with a lower risk of progression to HCC, in contrast to the F1b Basal strain, which predominates in Alaska and Peru [31,32].

In contrast, although the same genotypes were detected in OBI cases, F1b and A2 were the most prevalent, followed by F3. This finding differs from previous reports from Venezuela, in which subgenotype F2a was the predominant subgenotype among HIV patients with OBI [19]. Furthermore, earlier studies among Venezuelan blood donors associated occult infection with genotypes A and D [54], whereas most occult cases in Venezuelan Amerindians were attributed to genotype F3 [55], which is consistent with our current findings. Similarly, genotype F3 has been reported in both the general population and individuals with HIV and OBI in Colombia [56]. In Brazil, genotypes A1, A2, D2, D3, and F2 have been reported in patients with HIV, consistent with those described in the general population (A, D, and F) [51]. Altogether, these findings suggest that any HBV genotype is capable of establishing an occult infection; however, the prevalence of specific genotypes appears to depend on the circulating strains in a given geographic region and the risk factors to which the population is exposed to.

The overall HCV prevalence in the PLWH cohort evaluated was 4%, which is significantly higher than that reported in 2014 for the same cohort type (0.7%) [19]. However, this rate was lower than that reported in HIV-positive individuals from Latin America and the Caribbean (17.4%) [36], Brazil (11.5%) [57], and Mexico (36.4%) [58] but higher than that in Chile (1%) [35] and Colombian PWIDs (3.3%) [59]. Notably, while no cases of IDU were reported in the 2014 study [19], the marked increase in HCV prevalence in Venezuela might be linked to a rise in intravenous drug use in the country [60], consistent with WHO and CDC reports identifying parenteral transmission as the main route of HCV infection [61]. In a subgroup of the Venezuelan PLWH cohort with documented status regarding intravenous drug use (3.7%, 14/378), IDU was a significant risk factor associated with HCV infection. This observation aligns with recent large-scale evidence from vulnerable urban populations, where a history of injecting drug use was identified as the primary risk factor for HIV acquisition and was associated with a high active HCV prevalence among PLWH (15.7%) [62]. These findings underscore that IDU-driven HIV/HCV syndemics persist even in settings with broad ART access, reinforcing the need for integrated harm-reduction strategies and low-threshold care [62]. Beyond drug-use behavior, structural socioeconomic vulnerabilities, such as severe economic hardship, lack of income, and unstable housing, have been independently associated with an elevated risk of active HCV infection, alongside injecting drug use [63]. In Venezuela, where prolonged complex socio-sanitary conditions have impacted public health infrastructure and low-threshold prevention programs, HCV transmission is driven not only by IDU but also by structural socioeconomic determinants that limit access to prevention and care [20].

In the present study, the HCV prevalence among men was 5%, with no statistically significant sex-based difference (*p* = 0.295). No HCV serological markers were detected in any of the children or adolescents. Phylogenetic analysis revealed that HCV genotype 1a was the most prevalent, followed by genotypes 1b and 3a. These findings are consistent with previous Venezuelan reports [19] as well as global data on genotype distribution among PWIDs, where genotypes 1a and 3a have been the most prevalent [64]. Within this Venezuelan cohort, four HCV-positive individuals reported a history of PWIDs (carrying genotype 1a or 3a), reinforcing the hypothesis that increased intravenous drug use is driving HCV transmission in the country.

Chronic liver disease remains a major cause of morbidity and mortality in PLWH, and the observed trends in both HBV and HCV coinfections underscore the need for integrated screening and treatment strategies [4,6,7,9,34]. Although vaccination has reduced new infections in younger groups, active infection persists among older, probably unvaccinated adults. This highlights the importance of targeted screening and vaccination campaigns in at-risk adult populations in the future.

This study provides the first molecular epidemiology evaluation of HBV and HCV coinfections among PLWH in the Capital District of Venezuela in over a decade, evaluating complete HBV genomes, escape mutations, and resistance profiles in the context of current ART regimens. This is the first report linking HCV prevalence with intravenous drug use in the country and integrates both seroprevalence and robust molecular analyses, including NGS for whole-genome characterization. However, several limitations should be acknowledged. The cross-sectional design prevents establishing temporal causality, and critical clinical variables, such as exact vaccination coverage rates and duration of infection, could not be assessed. Finally, the study was restricted to a single urban center, which may limit the generalizability of the findings to other geographical regions in Venezuela.

## 5. Conclusions

In conclusion, this study demonstrates that active HBV infection is still a concern among PLWH in Venezuela over the past decade, whereas exposure has increased significantly in older adults and occult infection has declined, indirectly suggesting a positive impact of vaccination and probably the present ART treatment. The presence of vaccine-escape mutations in occult HBV cases highlights the risk of underdiagnosis and underscores the need for enhanced molecular surveillance. Furthermore, the notable increase in HCV prevalence, which might be associated with an increase in intravenous drug use, represents an emerging public health concern that must be addressed to achieve the eradication of this disease. These findings emphasize the critical importance of integrated screening programs, including molecular diagnosis for occult infections, continuous monitoring of viral genetic diversity and resistance mutations, and strengthened vaccination strategies to effectively control HBV and HCV coinfections in vulnerable populations.

## Figures and Tables

**Figure 1:**
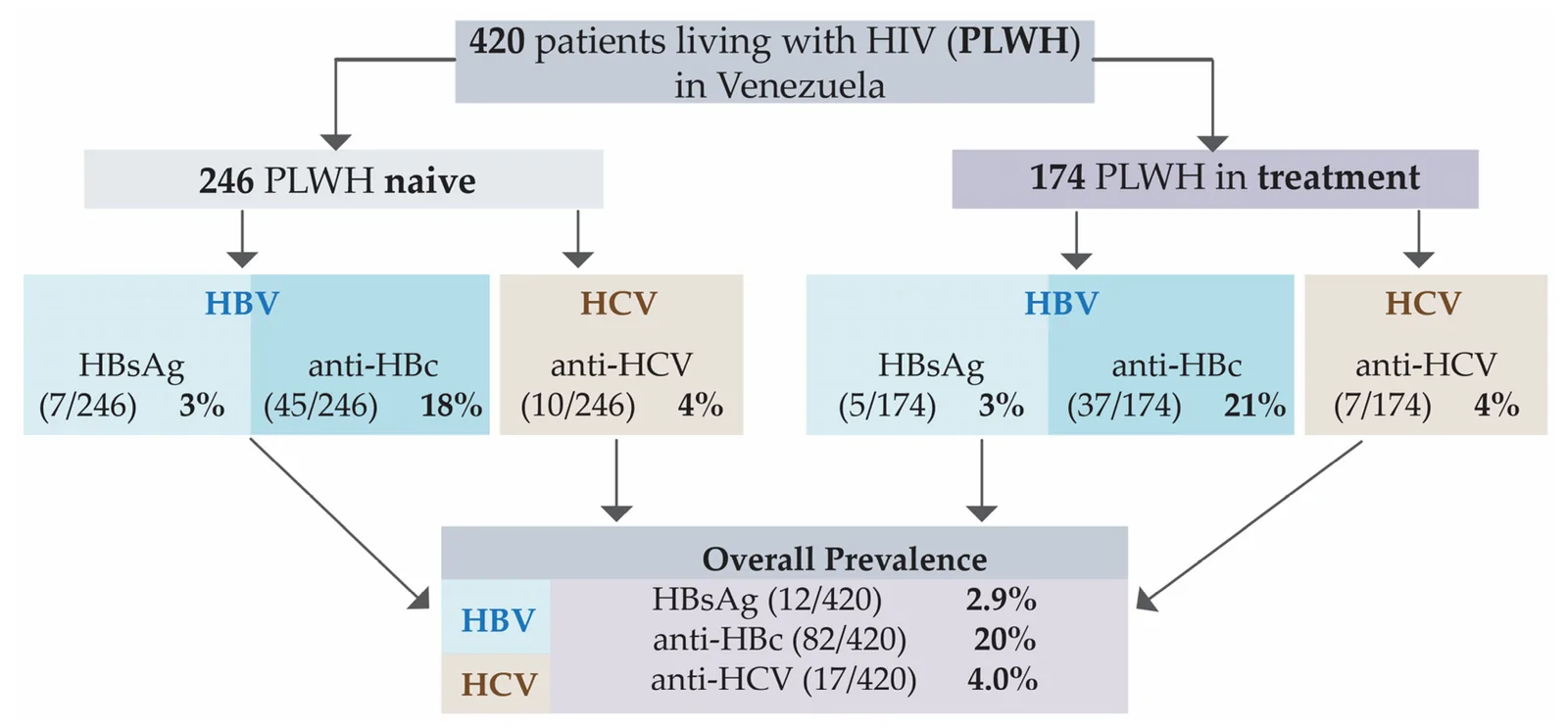
Seroprevalence of HBV and HCV according to the treatment status of individuals living with HIV in Venezuela.

**Figure 2:**
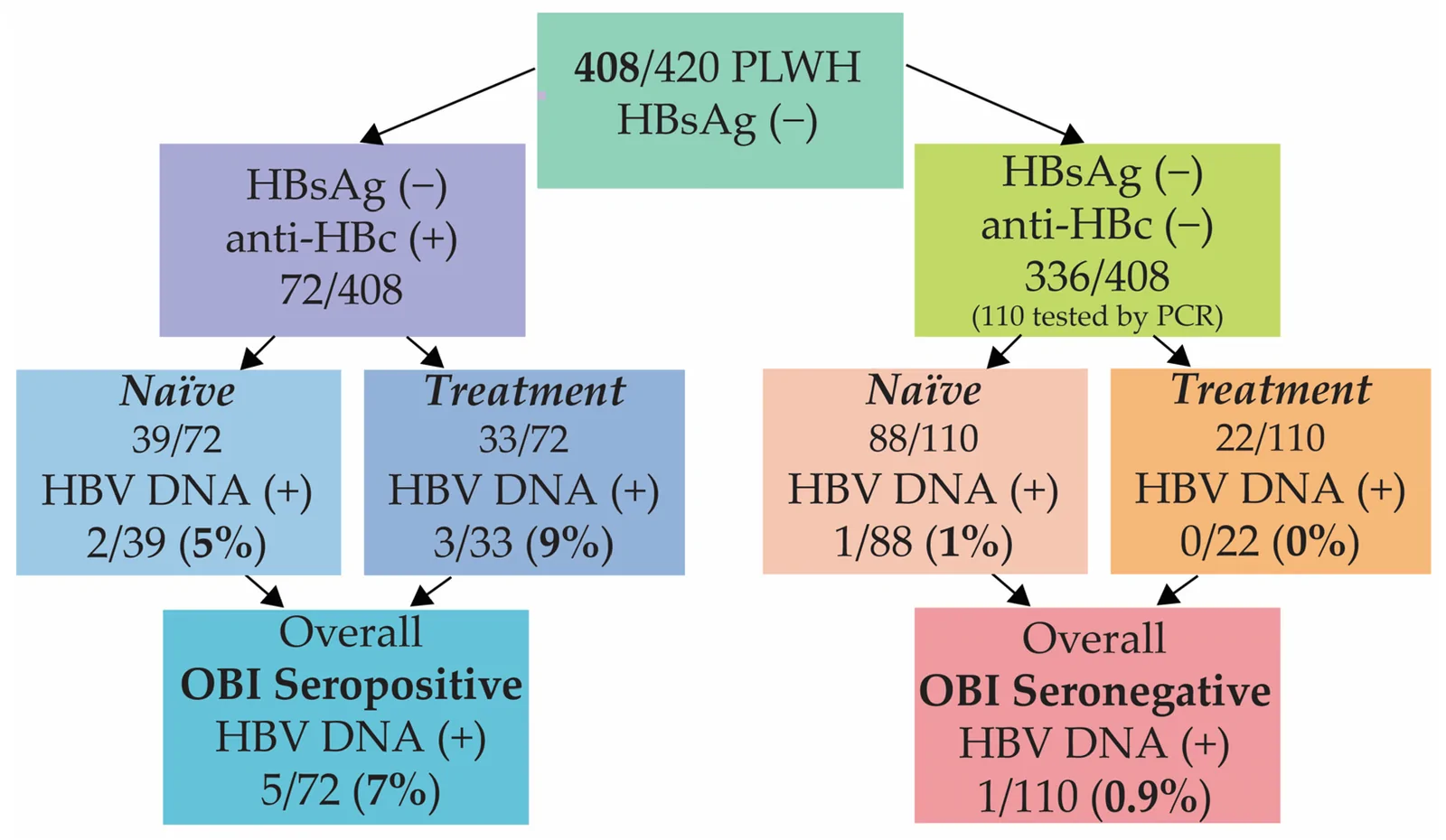
Occult hepatitis B infection (OBI) prevalence in PLWH. Positive and negative serological markers are represented by the + and − symbols, respectively.

**Figure 3:**
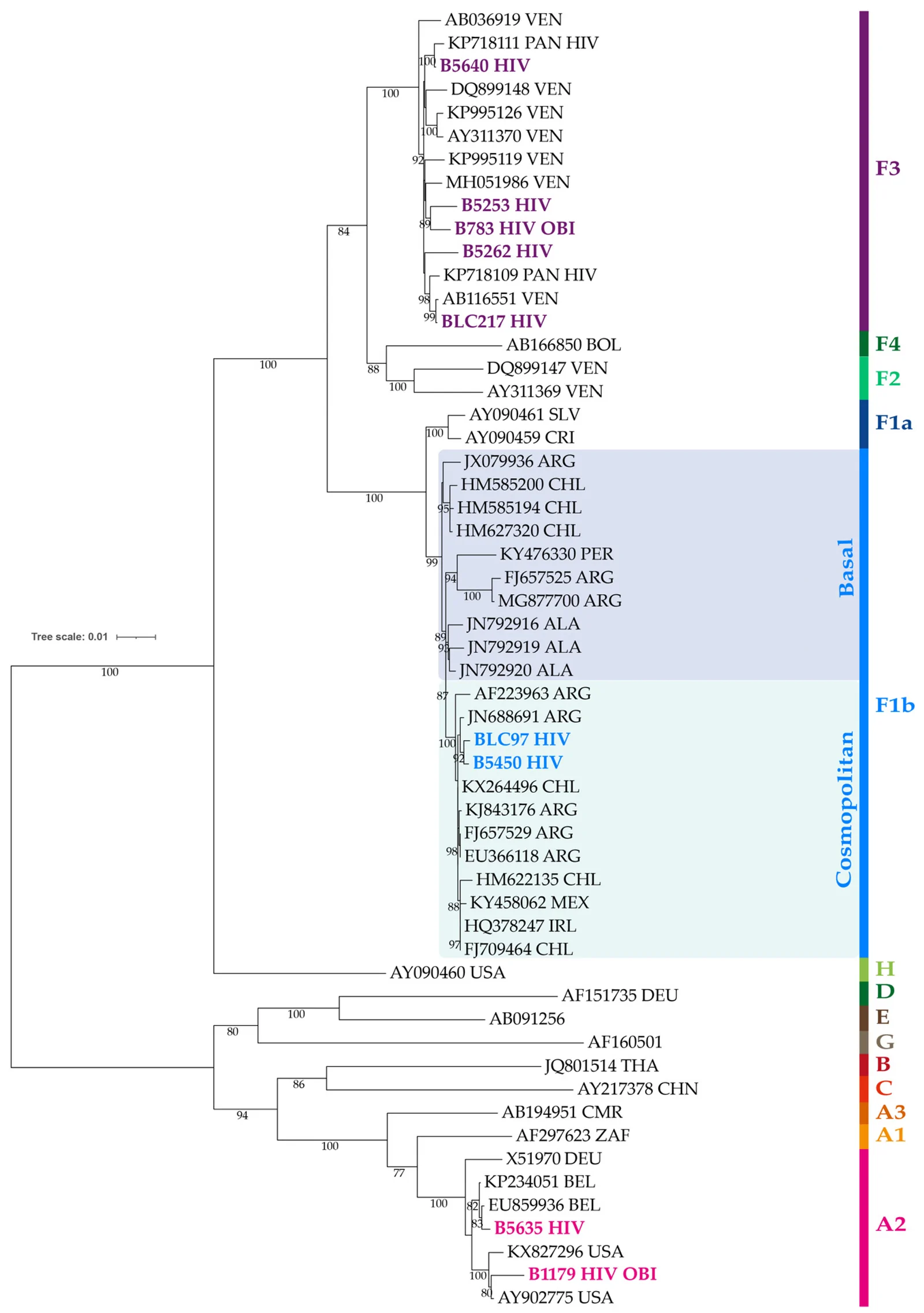
Phylogenetic tree of complete HBV genomes. Phylogeny was performed using the maximum likelihood method and transversion (TVM) nucleotide substitution model. Differences in the rate of evolution between sites were modeled using Empirical Base Frequencies (+F), with sites considered to be evolutionarily invariant (+I) and a free rate model with two categories (+R2). The number of each node corresponds to the bootstrap value obtained from 1000 replicates (bootstrap values greater than 70 are shown). Evolutionary analyses were performed on IQ-TREE. The right-hand bar represents the HBV genotypes, which are differentiated by different colors. Prototype strains from GenBank were designated according to accession numbers. The HBV isolates obtained in this study are highlighted in the corresponding genotype color. Shaded rectangles demarcate the Basal (upper) and F1b Cosmopolitan (lower) clades. Each entry includes the accession number, HBV subtype, and isolate country of origin. In some cases, information regarding HIV co-infection or occult HBV infection (OBI) was also included. VEN, Venezuela; PAN, Panama; BOL, Bolivia; SLV, El Salvador; CRI, Costa Rica; ARG, Argentina; CHL, Chile; PER, Peru; ALA, Alaska; MEX, Mexico; IRL, Ireland; USA, United States of America; DEU, Germany; THA, Thailand; CHN, China; CMR, Cameroon; ZAF, South Africa; BEL, Belgium.

**Figure 4:**
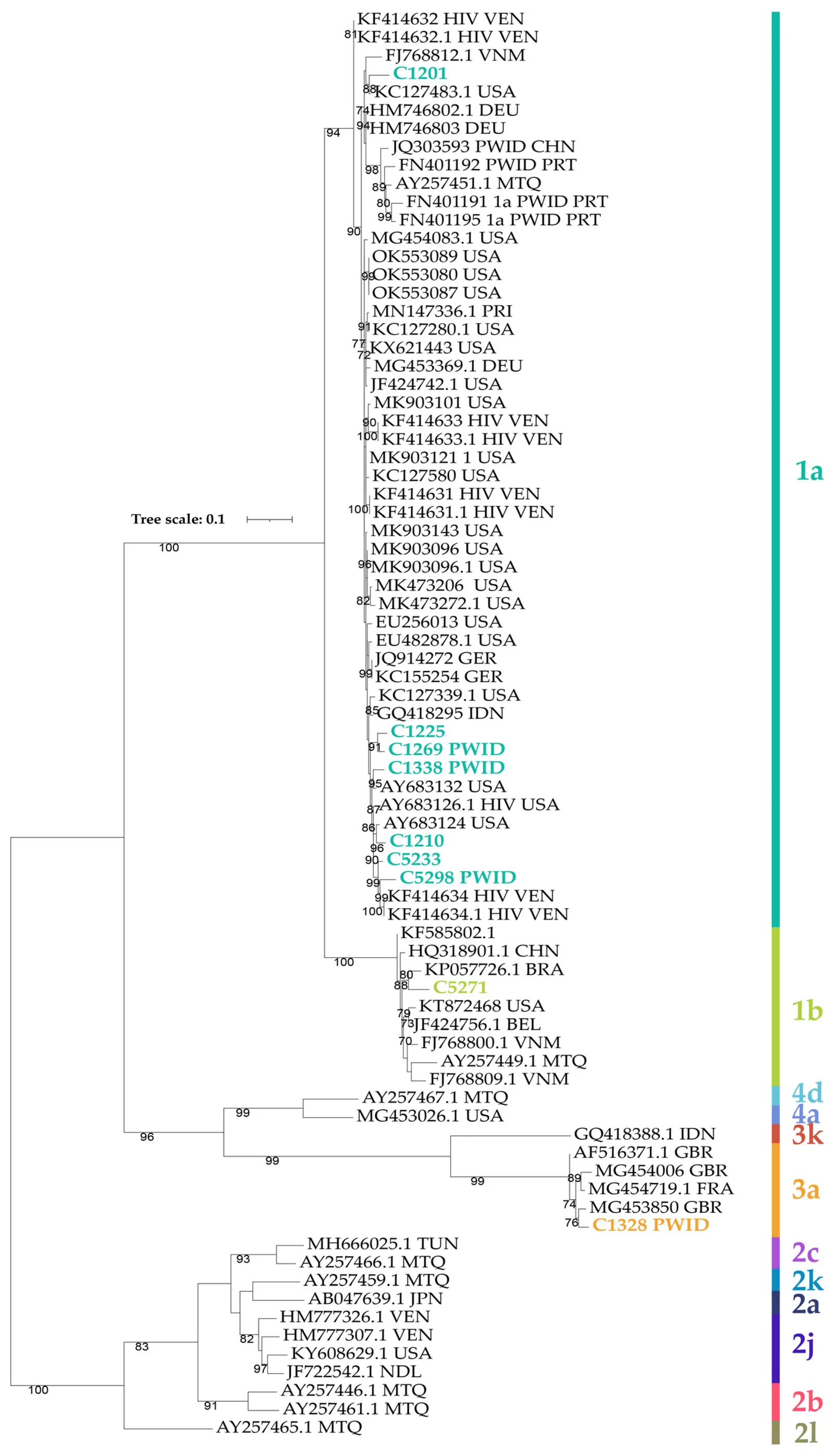
Phylogenetic tree of the HCV NS5B region. Phylogeny was performed using the maximum likelihood method and the TPM2u (Transversion Model 2 with unequal base frequencies) nucleotide substitution model. Differences in the rate of evolution between sites were modeled using Empirical Base Frequencies (+F), with sites considered evolutionarily invariant (+I) and a discrete gamma distribution with four rate categories (+G4). The number of each node corresponds to the bootstrap value obtained from 5000 replicates (bootstrap values greater than 70 are shown). Evolutionary analyses were performed using IQ-TREE. The right-hand bar represents the HCV genotypes, which are differentiated by different colors. Prototype strains from GenBank were designated according to the accession numbers. The HCV isolates obtained in this study are highlighted in colors corresponding to their genotypes. Each entry includes an accession number, country of origin of the isolate, and HCV subtype. PWIDs, people who inject drugs. VEN, Venezuela; VNM, Vietnam; USA, United States of America; DEU, Germany; CHN, China; PRT, Portugal; PRI, Puerto Rico; MTQ, Martinique; GER, Germany; BEL, Belgium; BRA, Brazil; IDN, Indonesia; GBR, United Kingdom; FRA, France; TUN, Tunisia; JPN, Japan; NLD, The Netherlands.

**Table 1:** Prevalence of serological markers against HBV and HCV in PLWH by sex and age group.

	Positive Serological Markers					
	HBsAg		Anti-HBc		Anti-HCV	
Age (Years)	F	M	F	M	F	M
0–10	0/8	0/4	0/8	0/4	0/8	0/4
11–20	0/17	0/20	1/17 (6%)	0/20	0/17	1/20 (5%)
21–30	0/20	1/80 (1%)	0/20	6/80 (8%)	0/20	5/80 (6%)
31–40	0/30	3/82 (4%)	4/30 (13%)	22/82 (27%)	1/30 (3%)	3/82 (4%)
41–50	1/34 (3%) *	1/56 (2%)	5/34 (15%)	20/56 (36%)	1/34 (3%)	2/56 (4%)
51–60	0/10	4/38 (11%)	1/10 (10%)	15/38 (39%)	1/10 (10%) *	3/38 (8%) *
61–70	0/7	2/13 (15%) *	2/7 (29%) *	6/13 (46%) *	0/7	0/13
Over 70	0/1	0/0	0/1	0/0	0/1	0/0
Total	1/127 (1%)	11/293 (4%)	13/127 (10%)	69/293 (24%)	3/127 (2%)	14/293 (5%)
Statistical difference	*p* = 0.117		*p* = 0.002		*p* = 0.295	

F, female; M, male. * Highest prevalence by sex and age group for each serological marker.

**Table 2:** HBV serological markers and viral loads in PLWH with overt infection.

	Sample ID	Sex/Age (Years)	ART Status	HBV Genotype	HBeAg	Anti-HBeAg	Viral Load in IU/mL
**HBsAg+** **anti-HBc−**	BLC97	M/28	Naïve	F1b	+	−	5.27 × 10^7^
	BLC110	M/68	TLD	−	+	−	0
**HBsAg+** **anti-HBc+**	BLC98	M/51	TLD	−	−	+	0
	BLC217	M/35	Naïve	F3	−	−	1.77 × 10^3^
	B1322	M/58	Naïve	NA	+	−	2.12 × 10^2^
	B5253	M/46	Naïve	F3	−	+	9.05 × 10^1^
	B5262	M/38	TLD	F3	+	−	4.26 × 10^4^
	B5450	M/40	Naïve	F1b	ND	−	1.43 × 10^8^
	B5580	M/64	TAF/FTC/BIC *	NA	+	−	3.47 × 10^1^
	B5600	F/49	TLD	−	+	−	0
	B5635	M/59	Naïve	A2	+	−	3.78 × 10^6^
	B5640	M/52	Naïve	F3	+	−	8.16 × 10^6^

F, female; M, male. TLD, tenofovir disoproxil fumarate/lamivudine/dolutegravir fixed-dose combination; TAF, tenofovir alafenamide; FTC, emtricitabine; BIC, bictegravir; * Additionally, the patient was receiving entecavir (ETV) for HBV infection. Positive and negative serological markers are represented with the + and − symbols, respectively. NA, not available for sequencing because the sample could only be amplified by qPCR; ND, not determined.

**Table 3:** HBV serological markers and viral loads in PLWH with occult infection.

	Sample ID	Sex/Age (Years)	ART Status	HBV Genotype	HBeAg	Anti-HBeAg	Viral Load in IU/mL
**OBI seropositive**	B783	M/33	Naïve	F3	−	−	5.38 × 10^1^
	B1058	F/52	TLD *	A2	−	+	3.06 × 10^2^
	B1179	M/68	Naïve	A2	−	−	1.56 × 10^2^
	B1304	M/32	Treatment *	F1b	+	−	1.43 × 10^2^
	B1331	M/63	TLD	NA	−	−	6.25 × 10^1^
**OBI seronegative**	BLC120	M/25	Naïve	F1b	−	−	8.82 × 10^1^

OBI seropositive, HBsAg-negative/anti-HBc-positive; OBI seronegative, HBsAg/anti-HBc-negative; F, female; M, male. TLD, tenofovir disoproxil fumarate/lamivudine/dolutegravir fixed-dose combination. * History of previous ART discontinuation. Positive and negative serological markers are represented with + and − symbols, respectively. NA, not available for sequencing because the sample could only be amplified by qPCR.

**Table 4:** HBV genotype distribution and prevalence among PLWH in Venezuela.

	F1b	F3	A2
PLWH with HbsAg+ (*n* = 7)	2/7 (29%)	4/7 (57%) *	1/7 (14%)
PLWH with HbsAg− (*n* = 5)	2/5 (40%) *	1/5 (20%)	2/5 (40%) *
Total (*n* = 12)	4/12 (33%)	5/12 (42%) *	3/12 (25%)

Positive and negative HBsAg results are represented by + and − symbols, respectively. * Most frequent genotype.

**Table 5:** HBV Mutation Analysis in S Protein.

						Detection		
Sample ID	Gender/Age/ART Status	HBV Infection	S Protein Mutation	Viral Load in IU/mL	Mutation Effect	ELISA 1	ELISA 2	Rapid Test
B5450	M/40/Naïve	Overt, F1b	118K	1.43 × 10^8^	IDE-VE	Yes	Yes	ND
B1304	M/32/Treatment *	OBI, F1b	120Q	1.43 × 10^2^	IDE-IE	No	No	No
B5262	M/38/Treatment *	Overt, F3	120Q	4.26 × 10^4^	IDE-IE	No	No	Yes
B1058	F/52/Treatment *	OBI, A2	144A	3.06 × 10^2^	IDE-VE	No	No	No

F, female; M, male. * Current ART regimen consists of TLD, with a reported history of previous ART discontinuation. OBI, occult HBV infection; IDE, immunodiagnostic escape; IE, immune escape; VE, vaccine escape. ELISA 1, Monocent ELISA kit; ELISA 2, Re-combi-LISA kit; Rapid Test, ARIA HBV-5 Rapid Test. ND, not determined.

## Data Availability

The complete HBV genome sequences were deposited in GenBank under accession numbers QB060664–QB060672. The S partial HBV gene sequences were deposited in GenBank under the accession numbers PZ863007–PZ863009. The HCV 5′UTR sequences were deposited in GenBank under accession numbers PZ862989–PZ862997. The HCV NS5B gene partial cds sequences were deposited in GenBank under accession numbers PZ862998–PZ863006.

## Abbreviations

The following abbreviations are used in this manuscript:

HBV	hepatitis B virus
HCV	hepatitis C virus
HIV	human immunodeficiency virus
HBsAg	HBV surface antigen
anti-HBc	antibodies against the hepatitis B core antigen
anti-HCV	antibodies against HCV
HBeAg	hepatitis B e antigen
anti-HBe	antibodies against HBeAg
ELISA	immunoenzymatic assay
5′NC	5′ non-coding region
OBI	occult hepatitis B virus infection
IDU	injection drug use
PWIDs	people who inject drugs
